# About Cattle Ticks

**Published:** 1892-01

**Authors:** Cooper Curtice

**Affiliations:** Veterinarian, Moravia, N. Y.


THE JOURNAL
OF
COMPARATIVE MEDICINE AND
VETERINARY ARCHIVES.

VOL. XIII. JANUARY, 1892. NO. 1.

### ABOUT CATTLE TICKS.

BY COOPER CURTICE, M. D., VETERINARIAN,
Moravia, N. Y.

In the July number of the last volume I described how the young tick hatching from the so-called egg, passed through a six-legged and an asexual eight-legged stage to again moult before emerging as adult ticks.

The differences between the ticks destined to become either male or female during their final moult is not marked. The average of the males is smaller, but a small female may not be any larger than an ordinary male. In each the mouth ring and mouth parts, the shield-like head-piece, the breathing pores, the limbs and the body are alike.

After they emerge, however, the males can be quickly chosen by their smaller size, by the absence of a well-defined head shield, by the extension of the shield over entire back, and by the two pairs of triangular chitinous plates, situated on the abdomen, behind and on each side of the anus. The female looks much as in her earlier stage; the head shield is, however, larger and stronger, the lines made by the muscular attachments to the body-walls are stronger and deeper, and the breathing pores are much enlarged. The limbs, in both male and female, are strong and large as compared with their bodies, and fit them for retaining their place on their host until they have gained a new attachment by their mouth or rostrum. The external genitals which appear in the adults are very similar in each sex, and occur between the bases of the second pair of legs. They present little more than an opening situated on the middle line of the belly.

Throughout life the male enlarges but little; he becomes a little broader, longer and thicker, but not markedly so. The female, on the contrary, grows to a comparatively immense size, swelling day by day, her body becoming so rotund and replete with the food drawn from her host that she can scarcely be recognized as of the same species as the males. While her body has inflated, however, her head, her legs and breathing pores have not undergone any changes. These remain exactly of the same size as in the beginning, and with the exception of the head shield are but little different from those of the male. The disparity in size between the legs and the body of the fully-gorged female is so marked that the legs and head appear even smaller than at first. The comparatively small size of the male has caused it to be overlooked or, if found, caused it to be classed among the young of this species.

After moulting, the young female again attaches herself to her host, and seems rarely to change her position. While she may be able to do so at first with ease, she becomes so heavy and logy later on that any change would cause her to fall to the ground should she loosen her hold with her beak. The males, however, remain small and light, and it is not impossible for them to change their position, and no doubt do so. After moulting they hunt for mates through the dense growth of cattle hairs and, finding them, attach to their host so that they can conveniently embrace them belly to belly, and bring their external genitals in opposition. In this position the males may be found with females of all sizes. That the attachment of the male is for food, as well as for copulation, there can be but little doubt, for the long continuance of life, the increase in size, and the tremendous drain upon the little fellow in fertilizing the eggs, demand it.

Ticks live upon the blood of their hosts. The female rapidly increases in size, storing away quantities of the ingested food in an immense convoluted chamber which may be likened to a liver. The rapidity with which she enlarges seems to depend much on temperature; in summer three or four days only are necessary for the production of quite large ticks after the final moult. The blood eaten is stored as a dark colored fluid which coagulates in alcohol. I have seen no blood corpuscles in it.

While ingesting the blood and assimilating it the tick may, and probably does, digest it to some extent. I have not demonstrated a stomach or accessory organs however, not having made necessary microscopic sections.

Underneath the skin of the back of living females can be traced certain tortuous vessels, one on each side, which contain a white fluid substance. This also becomes solid after death in alcohol. These vessels are of the excretory system and empty with the intestines at the anus. The presence of this material also demonstrates a digestion of food and a waste product in the living processes of the animal.

Breathing takes place through the two pores with sieve-like plates, situated at each side of the body, just behind and above the last pair of legs. From the main opening the air is distributed by a fascicle of tubular branches (tracheæ), into all parts of the body.

After vegetation the most interesting function in ticks is reproduction. The male places himself in copulation as noted above, belly to belly with the female, attaches to the host by his beak and winds his legs around those of the female, thus bringing their external genitals in contact. This is always easily effected, for whatever be the difference in size between them, the distance from the point of the beak to the genital opening in each is nearly equal. In each the genital opening is about as far behind the mouth-ring as the beak and ring is long.

It is not probable that the male inserts the penis into the female. It is quite likely that each may protrude their parts until they meet. While they touch the semen must pass from the male to the female. From the continued presence of the male by the female one would infer that copulation was not completed at once, but rather through a series of connections more or less remote.

The internal apparatus of the female consists of paired ovaries, uteri, and a single vagina or ovipositor. There are other accessory organs among which is a receptacle for storing semen. The ovaries, of course, supply the eggs which pass into the uteri to become fertilized, take on shells and become otherwise changed. The uteri though comparatively large do not occupy as much of the body cavity as one would think from a glance at the mass of eggs laid ; for before the tick loosens her hold from the cow but few are developed, and during ovipositing, conversion of the stored food and corresponding development of eggs take place with tremendous rapidity.

When fully gorged, when the organs of generation are fully prepared, and either the eggs within fertilized or a sufficient quantity of semen stored in the receptacle for their fertilization, the female loosens her hold on her host and falls to the ground. She must do this to lay her eggs. Crawling off to some dark corner her work soon begins. Any delay seems to me to be caused by the tick not being prepared to undergo the final act at the time of removal from the cow. The female may, if detached, lay eggs any time after it is half grown. Most ticks under my observation have waited a day or two before commencing ovipositing, and some even more. While the tick prefers to act in quiet she will, if retarded long enough, show her secret method under almost any difficulties.

I must now draw attention to an organ which, though accessory, plays an important role in ovipositing. Between the mouth ring and the head shield, is a space which becomes very marked in the fecund tick ; at this point open glands, which are paired, racemose and situated just under and within the head shield. During the last days of the growth of the tick these glands become distended with a viscous fluid substance with which the eggs are to be coated for protection.

The first visible act in ovipositing is the withdrawal of the mouth ring and appendages apparently into the body, thereby leaving a depression or pocket; at the same time the ovipositor protrudes toward the bulging skin at the back of the mouth ring until they touch. The head is now entirely concealed. As soon as the ovipositor touches the opposing organ at the slit which appears in its middle, an egg passes from it and is immediately surrounded by the coating sac. This passage of the egg is difficult to detect, but if the passage is interfered with can be made out after a time The ovipositor then withdraws, the mouth parts appear, and the egg is pushed from its coating sac which recedes from around it. As the mouth parts are commonly known as the head, it appears as though the female passed the egg over her head and laid them from her neck. A curious affair surely.

The object of coating the egg has been clearly demonstrated by a German, who found that eggs laid after destroying the coating sac and preventing the eggs being covered, dried up and would not hatch, while others laid by the same female and coated, hatched in due time. Egg after egg does the little creature lay, her pile growing constantly larger while her body constantly contracts, until, in about a week, little is left but a yellowish, dried up, shriveled skin, whence all life has departed. She dies, having nothing else to live for ; her young, when hatched, need neither care nor direction. Sufficient of them will find a cow and live again through all the phases of their life's history, and their posterity be quite sure to pester the future farmer and investigator.

For the cattle tick, these notes and observations are original. One and another investigator has proved some of the facts for other European ticks, but even yet many of the statements made are contested in print throughout various articles, while but one investigator has described copulation and oviposition correctly.

To rid cattle of ticks is no easy task, especially when the former go daily upon the pastures. To keep them free even when standing in the stall or barnyard is equally difficult, especially in the South, where the tick seems to persist the year 'round, excepting, possibly, a short period in the winter.

Still, treatment is productive of good, and should be practiced. Almost any farmer in the tick area will ask the investigator why it is that some cattle are literally laden with ticks while others are free, or nearly so. It is usually the fat cattle that enjoy the most immunity. Whether the ticks thrive better on poor cattle or having attacked an animal causes it to become poor is a debateable question. I believe that each proposition is true, and that the fat in an animal's skin, or the oily condition of the hide, has much to do in protecting it against extensive invasion by ticks. It follows, therefore, that cattle should be kept in good order to resist these pests, as well as other diseases.

Another phase must be discussed. Cattle, on their feeding grounds, usually towards noon, seek favorite resting places, be they under spreading trees, by some fresh pool of water or upon the mountain top. Some of them continually return to the same spot day after day. Now, if some of them become infected in the course of feeding or resting, and these particular animals continue in their habit day by day, we can understand how they become more and more infected from the young ticks they themselves have introduced into their resting places, while others of their mates which have escaped from the first continue unmolested. So, too, after a tick stricken animal has been introduced into a stall and kept there a week, a month or longer, it continually becomes more and more infested, and perhaps more than it would in pasture. I have seen a bull kept under just such conditions that was litterally shingled with young ticks.

Dr. M. Francis, veterinarian of the A. and M. College, College Station, Texas, once wrote me that the kerosene emulsion advised worked to a charm. I am of the opinion that even a weak solution of this emulsion would be beneficial in keeping young ticks from the cattle if applied periodically. With dairy cattle its use might prove objectionable. When first used to kill ticks it should be thoroughly applied preferably with a spray nozzle, and especially to the nether parts of the cattle, those parts the ticks most frequent. I also believe that milch cows can be protected by oiling the limbs, the breast, the abdomen and. the escutcheon with sweet or cotton seed oils, or with their emulsions, with no damage. I have not tried this, but believe the remedy feasible, not only on account of the ticks, being liable to have their breathing pores stopped, and of their supposed aversion to oils, but on account of the difficulty they will have in progressing among the oily, sticky hairs.

That ticks have something to do with the "Southern cattle fever," has long been the popular opinion of many who have observed the two going hand in hand. That ticks may spread the fevers has been quite thoroughly demonstrated by the laboratory experiments of the Bureau of Animal Industry. How newly born ticks, that never have been on a diseased animal, may do this, has not been made quite so clear. We may suppose that a tick which has sucked the blood of a diseased host may loosen, and, getting on another animal in some way inoculate it; but not so with the newly hatched, unless the disease germs lay within the egg before leaving the parent. As both adults and young produce the fever, the necessity of preventing their spread on to Northern pastures, either in or at the end of transit, is pointed out, thus preventing one means of the spread of the fever. This, perhaps, is the most practical point that can be made in the study of this parasite.

If, therefore, the State and the United States authorities may have permitted cattle carrying ticks to be shipped into the area north of the "Southern cattle fever" area, which I believe they should not, those receiving them should attend to it that such cattle are cleansed of their ticks, and that the ticks be destroyed at once. As old ticks may drop from cattle into cars while en route and the young hatch there, the cars should be cleansed from the ticks as much as from the supposed germs of the "Southern cattle fever," that other cattle transported soon after may not get them.

The killing of ticks on all the cattle of a farm and preventing more getting on again should lessen the number for next year. Where there are no fences the results are not so sure, for cattle roaming everywhere pick up more. It is not positively known that cattle are the only animals which harbor these ticks. Indeed, I suspect that a small proportion may get on other animals. Dr. Francesco de Balmaseda, of Havana, Cuba, has sent me specimens which look like the cattle parasite and came from a dog. There were, however, no well developed adult females among them. If other animals should be found to harbor them, the supply from this source would be inconsiderable as compared with those cattle get from each other. Keeping one's cattle free from ticks should, therefore, so free the pastures that what at first would seem a formidible task would grow easier and easier. But persistent effort, accompanied by watchfulness, is needed.

